# Strength plus Endurance Training and Individualized Diet Reduce Fat Mass in Overweight Subjects: A Randomized Clinical Trial

**DOI:** 10.3390/ijerph17072596

**Published:** 2020-04-10

**Authors:** Pedro J. Benito, Bricia López-Plaza, Laura M. Bermejo, Ana B. Peinado, Rocío Cupeiro, Javier Butragueño, Miguel A. Rojo-Tirado, Domingo González-Lamuño, Carmen Gómez-Candela, on behalf of the PRONAF Study Group

**Affiliations:** 1 LFE Research Group, Department of Health and Human Performance, Faculty of Physical Activity and Sport Science-INEF, Universidad Politécnica de Madrid, 28040 Madrid, Spain; anabelen.peinado@upm.es (A.B.P.); rocio.cupeiro@upm.es (R.C.); javier.butragueno@gmail.com (J.B.); ma.rojo@upm.es (M.A.R.-T.); 2 Department of Nutrition, Hospital La Paz Health Research Institute (IdiPAZ), La Paz University Hospital, 28046 Madrid, Spain; bricia.plaza@idipaz.es (B.L.-P.); laura.bermejol@salud.madrid.org (L.M.B.); carmengomezcandela@telefonica.net (C.G.-C.); 3 Laboratory of Pediatrics, Department of Medical and Surgery Sciences, Faculty of Medicine, University of Cantabria, Marqués de Valdecilla Biomedical Research Institute (IDIVAL), 39011 Santander, Spain; gonzaleld@unican.es

**Keywords:** physical activity, endurance training, strength training, dietary modification, body composition

## Abstract

Studies with overweight people are a priority in order to observe the effect of the timing of intervention on pre-obesity people. The aim was to compare different physical activity programs plus an individualized hypocaloric diet on body composition in overweight subjects. A randomized controlled clinical trial was carried out in overweight adults with no history of relevant illness. Primary outcome was total fat mass (TFM). Participants were allocated into four activity programs with equal intensity and volume of exercise for 22 weeks: strength training (S), endurance training (E), strength + endurance training (SE), and ‘adhering to physical activity recommendations’ (C). Participants followed a diet with 25% less energy (50%–55% carbohydrates, 30%–35% fat) measured by accelerometer. Variables were assessed at baseline and at the end of the intervention. Body composition was measured by dual-energy X-ray absorptiometry. One hundred nineteen from 205 subjects were randomized in the four exercise groups (S = 30/E = 30/SE = 30/C = 29) and 84 participants (36 men/48 women) ended the intervention (S = 19/E = 25/SE = 22/C = 18). At the end of the experiment, all groups except C increased their total physical activity (S = 1159 ± 1740; E = 1625 ± 1790; SE = 1699 ± 2516; C = 724 ± 1979 MET-min/week). Using an ANOVA-test, improvements were observed in body weight (S = −4.6 ± 4.5; E = −6.6 ± 4.6; SE = −8.5 ± 2.8; C = −6.1 ± 5.6 kg, *p* = 0.059) and TFM (S = −4.24 ± 2.02; E = −4.74 ± 2.96; SE = −6.74 ± 3.27; C = −3.94 ± 4.18%; *p* < 0.05). The main conclusion was that there were no adverse events. Strength and endurance training with a balanced, individualized hypocaloric diet was the most effective at reducing weight loss and fat mass in overweight subjects. Trial registration: NCT01116856.

## 1. Introduction

The majority of epidemiological studies indicate that an elevated body weight during midlife is associated with an increased risk of all-cause mortality [1,2,3]. People with a body mass index (BMI) of 25–28.9 kg/m^2^ are at twice the risk of developing cardiovascular disease as people with a BMI of 18–21 kg/m^2^, while those ≥29 kg/m^2^ are at almost three times the risk [4]. Further, the Framingham Heart Study showed that being overweight at the age of 40 reduces life expectancy by three years [5]. In Spain, the overweight prevalence in adults aged 18–60 years is 34.2% (20.6% men, 13.6% women) while obesity prevalence in the same age range is 13.6%. Moreover, 70.2% of people have excess body fat and 54.7% have high cardiovascular risk (waist/height ratio of ≥0.5) [6]. 

Physical activity is widely recognized as a means of preventing chronic disease [7]. Increasing physical activity and the following of a balanced diet are crucial in any program to combat overweight and obesity [8,9,10,11,12]. It has recently been shown that, when combined with a hypocaloric diet, keeping to physical activity recommendations is just as effective as following an exercise training program to achieve a reduction in body weight and body composition in obese subjects [13]. However, it has not been reported whether this holds true for overweight subjects whose physiological and endocrine responses may differ when following weight loss programs [14,15]. 

The overweight population is at increased risk of developing the comorbidities of obesity [16]. Investigating weight loss strategies is, therefore, important. Evidence exists that undertaking endurance (i.e., aerobic) exercise while following a very restrictive energy intake (<50%) can reduce body weight [17,18]. However, data are scarce regarding the effect on weight loss of combining endurance and strength training with a much less restrictive, and therefore more sustainable, hypocaloric diet [17]. Those trials that have been performed have varied in terms of intensity, delivery method, target groups, and study components; the comparisons that can be made among them, therefore, are limited. It has thus been difficult to confidently design evidence-based physical activity interventions for overweight subjects [19,20].

Therefore, the aim of the present study was to compare the effects of different physical activity programs, in combination with a balanced, individualized hypocaloric diet, on anthropometric and body composition variables in overweight subjects, as part of the Nutrition and Physical Activity Programs for Obesity Treatment (PRONAF) project. This project was created taking into account the Global Strategy on Diet, Physical Activity and Health from the WHO, where health should be protected and promoted by guiding the development of an enabling environment to reduced disease and death rates related to unhealthy diet and physical inactivity [21]. The PRONAF project developed two randomized clinical trials to elucidate what strategy is the most effective for overweight and obesity treatment. The present work focuses on comparing the effects of different physical activity programs in combination with a balanced and individualized hypocaloric diet on body weight and body composition in overweight subjects. 

## 2. Materials and Methods 

### 2.1. Study Design

A parallel, randomized controlled trial was carried on in healthy men and women for 22 weeks. A CONSORT checklist is provided as an additional file. Subjects were selected by the Facultad de Ciencias de la Actividad Física y del Deporte (INEF) researchers. Subjects who initially fulfilled the inclusion criteria and passed a baseline physical examination (*n* = 119) were stratified by age and sex in blocks using a randomisation table provided by the Biostatistics Department of HULP. Then, they were assigned to interventions: strength training group (S = 30), endurance training group (E = 30), strength + endurance training group (SE = 30), or ‘adhering to physical activity recommendations’ group (control; C = 29). Additionally, all participants were prescribed a balanced, individualized hypocaloric diet. Baseline measurements were made at the beginning and at the end of the intervention period (48–72 h after last exercise session). 

### 2.2. Participants 

Detailed information on the materials and methods has been previously reported [22]. Participants were recruited via advertisements posted in newspapers and announced on the radio, Internet, and TV (September–October, 2009). The eligible sample consisted of 119 overweight subjects (73 women/46 men) living in Madrid, Spain. The inclusion criteria included ages ranging from 18 to 50 years, overweight (BMI ≥25–<30 kg/m^2^), normoglycaemic (glucose <100 mg/dL), non-smokers, sedentary lifestyles (<30′ physical activity/day), no medications or drugs use, no history of important illness (heart, lung, or liver disease or neoplasms), and regular menstrual cycles in females. The exclusion criteria covered all physical and psychological diseases that may have precluded the performance of the requested strength or endurance training, along with the taking of any medication known to influence physical performance or that might interfere with the interpretation of the results. 

The volunteers who fulfilled the inclusion criteria and passed the baseline physical examination were stratified by age ranges and sex and randomized into the different intervention groups.

In agreement with the Declaration of Helsinki, all participants signed a document of informed consent. The study was approved by La Paz University Hospital’s (HULP [HULP Code PI-643]) Ethics Committee. This trial was retrospectively registered as NCT01116856 at http://clinicaltrials.gov/. This manuscript does not contain any individual person’s data.

### 2.3. Exercise Interventions

The exercise training for S, E, and SE groups was 3 times/week. The intensity of (percentage of 15 repetition maximum (RM) in strength groups or percentage heart rate reserve (HRR) in endurance groups) and volume of exercise for these three groups was designed to be equal [13,22]. All training sessions were made in the INEF installations and supervised by certified personal trainers. The S group included shoulder presses, squats, barbell rows, lateral splits, bench presses, front splits, biceps curls, and French presses for triceps. Running, cycling, or elliptical exercises were included for the E group, while the SE group followed a combination of cycle ergometry, treadmill, or elliptical exercises intercalated with squats, rowing machine, bench presses, and front split exercises (15 lifts per set or 45″ SE endurance phase). The C subjects were encouraged to following general recommendations of the American College of Sports Medicine (ACSM). Thus, they undertook to exercise for at least 200–300′ of moderate-intensity physical activity/week (30–60 min/day) [23]. They were also advised to reduce their sedentary behaviour (e.g., watching television) and increase daily activities such as brisk walking or cycling instead of using a car, etc. [24].

The exercise training programs were designed taking into account each subject’s muscular strength (MS) and HRR. MS was measured in the S and SE subjects using the 15-RM testing method every other day during the week before the intervention period. The HRR was calculated to set the exercise intensity (50%–60% maximum heart rate-resting heart rate) for the E and SE subjects. The volume and intensity of the three training programs were equal and increased progressively during the study. In weeks 2–5, exercise was at an intensity of 50% of the 15-RM and HRR, and lasted an overall 51′ and 15″. In weeks 6–14, exercise was performed at an intensity of 60% of 15-RM and HRR, again with duration of 51′ and 15″. Finally, in weeks 15–22, exercise was performed at an intensity of 60% of 15-RM and HRR, with a duration of 60′. Each training session for the S, E, and SE subjects commenced with a 5′ aerobic warm-up, followed by the main session exercises, and concluded with 5′ of cooling down and stretching exercises. In all sessions, the exercise rhythm was controlled by instructions recorded on a compact disk. The energy expenditure during the training sessions was controlled by individualized heart rate vs. VO2 regression analysis. 

### 2.4. Hypocaloric Diets

Balanced, hypocaloric diets (between 1200 kcal (5020 kJ) and 1850 kcal (7732 kJ)) were prescribed individually for all participants by expert nutritionists at the Department of Nutrition, HULP, Madrid. The diet was designed to provide 25% less energy than baseline total daily energy expenditure (DEE), as measured using a SenseWear Pro Armband accelerometer (Body Media, Pittsburgh, USA) (reliability in the two resting visits r = 0.93; *p* < 0.001) [25]. The diet provided 30%–35% of energy from fat, and 50%–55% from carbohydrates, according to the recommendations of the Spanish Society of Community Nutrition (SENC) [26] and 20% from protein to achieve the body composition benefits observed in different studies and examined in a recent meta-analysis [27]. The hypocaloric diet program was followed during the 22-week intervention period. Dietary counselling was given at baseline and at 12 weeks to resolve questions and to motivate participants sufficiently to comply with dietary advice. All subjects were instructed on how to record their dietary intake using a daily log and given recommended portion sizes and information on possible food swaps. In addition, there were five nutrition education group sessions during the study given by dieticians where all participants were encouraged to follow the diet prescribed. The goal was to equip the participants with the knowledge and skills necessary to achieve gradual but permanent behaviour changes. 

### 2.5. Analytical Methods, Measurement of Body Composition, Dietetic, and Physical Activity Variables

The following analyses and measurements were made at baseline and at the end of the intervention period. All data were collected by the Department of Nutrition, HULP.

#### 2.5.1. Weight and Body Composition Variables

Height was measured using a SECA stadiometer (range 80–200 cm). Body weight was measured using a TANITA BC-420MA balance (Bio Lógica Tecnología Médica S.L, Barcelona, Spain). The BMI was calculated as (body weight (kg)/(height (m^2^)). Waist circumference (WC) was measured using a SECA 201 steel tape (Quirumed, Valencia, Spain). Dual-energy X-ray absorptiometry (DXA) was used to measure the total fat mass (TFM), android fat (AF), android/gynoid fat ratio (AF/GF), and total lean mass (TLM), employing a GE Lunar Prodigy apparatus (GE Healthcare, Madison, Wisconsin, USA). All DXA scans were performed using GE Encore 2002 software v.6.10.029.

#### 2.5.2. Dietetic Study

All food and beverages consumed by the participants were recorded using a food frequency questionnaire and a “3-day food and drink record”, for a Spanish population. A 24-h food recall was used as a dietetic control at 12 weeks. At the beginning of the study, participants were instructed to record the weights of food and beverages consumed using a kitchen scale whenever possible, and to use household measurements (tablespoons, cups, etc.) when not. Queries about the diet daily fillings were resolved by a trained researcher (dietician). When questionnaires were returned by the subjects, they were reviewed by the trained dietician and the subject to identify possible filling mistakes and omissions (mealtimes, foods, weights, etc.). The food energy and nutritional content consumed were calculated using DIAL software (Alce Ingeniería, Madrid, Spain). The Healthy Eating Index (HEI) was calculated according to Kennedy et al. [28], taking into account the number of servings recommended for the Spanish population [26]. Compliance with recommended intakes was assessed for the different food groups (cereals, vegetables/greens, fruits, dairy products, and meat/fish/eggs, expressed in servings/day), and for meeting nutritional objectives (intake of lipids, saturated fatty acid, cholesterol and sodium, and dietary variety). Each of these 10 factors was awarded a maximum of 10 points when the intake was the same as that recommended and a minimum of 0 points when the difference was very great. Intermediate values were awarded proportionally. Diet quality was deemed “good” when more than 80 total points were scored, as “needing improvement” with a 51–80 score, and “poor” with a score below 50 [28]. 

#### 2.5.3. Physical Activity Variables 

Physical activity was assessed using seven items in the International Physical Activity Questionnaire-Short Form (IPAQ-SF), which asks about the frequency and duration of vigorous intensity activity (1 and 2 items), moderate intensity activity (3 and 4 items), and walking activity (5 and 6 items). The questionnaire was scored using established methods posted on the IPAQ website (www.ipaq.ki.se). The questionnaire took approximately 8–10′ to complete; a trained researcher assisted participants with answering. The collected data were summarized to classify the subjects into high, moderate, and low physical activity groups. Time spent in vigorous, moderate, and walking activity was weighted by the energy expended for these categories of activity, to produce the total MET-minutes of physical activity per week (MET-min/week). In addition, the IPAQ-SF contained an indicator item of time spent in sedentary activity (item 7): “During the last 7 days, how much time did you usually spend sitting on a weekday?” Sitting time/week was estimated taking into account the answers supplied.

Cardiovascular fitness was evaluated via a maximal stress test using the modified Bruce protocol [29] and a computerized treadmill (H/P/COSMOS 3PW 4.0, H/P/Cosmos Sports & Medical, Nussdorf-Traunstein, Germany). Peak oxygen consumption (VO2peak) was measured with a Jaeger Oxycon Pro gas analyser (Viasys Healthcare, Höchberg, Germany). The stress test was continued until exhaustion. The mean of the three highest VO2 measurements was taken as the VO2peak value. 

### 2.6. Adherence to Diet and Exercise

Adherence to diet was calculated as (estimated energy intake/real energy intake) × 100. Complete adherence = 100%; high values reflect greater dietary restriction to the estimated energy intake, lower values, lesser restriction. Those participants who failed to meet 80% adherence were excluded from further analysis. 

Adherence to exercise was calculated as the number of sessions completed with respect to the theoretical maximum (sessions completed/total number of sessions possible) × 100. An adherence of 90% was demanded for continued inclusion.

### 2.7. Statistical Methods

The TFM was used to calculate the expected effect size: 1.96 for C group and 2.18 for the experimental groups. The sample size was calculated to detect the effect of training and diet on TFM as the primary outcome (TFM), with 80% statistical power, and with significance set at *p* < 0.05, assuming a correlation of 0.80 between repeated measures, and assuming an estimated drop out of 20%. 

All analyses were performed using SPSS v.17.0 software (SPSS Inc., Chicago, IL, USA). Significance was set at *p* < 0.05. 

The Kolmogorov–Smirnov test was used to determine whether data were normally distributed. Means and standard deviations (SD) were calculated for continuous variables. Two-way analysis of variance (ANOVA) with repeated measures was used to determine differences among the four interventions groups and between baseline and post-training values. Multiple ANOVA comparisons were made employing the Bonferroni post hoc correction. The Chi-squared test was used to examine the relationship between quantitative variables.

## 3. Results

**Figure 1:**
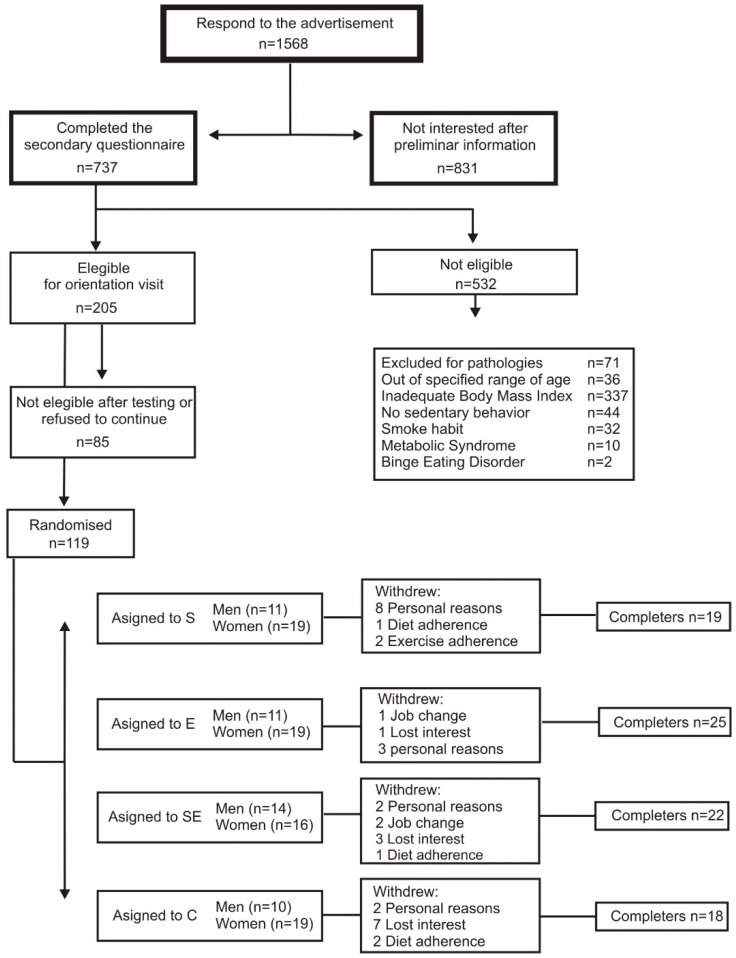
Participant flow diagram. N = 84, 48 women/36 men.

The clinical study was carried out from January to June (2010). Of the 119 subjects randomized to the four interventions (S = 30; E = 30; SE = 30; C = 29, 73 women/46 men), 84 completed the clinical trial and were included in the statistical analysis (S = 19; E = 25; SE = 22; C = 18 with 48 women/36 men). Participant withdrawals were mainly due to personal reasons or loss of interest (Figure 1). 

### 3.1. Baseline Characteristics 

The baseline characteristics of the 84 participants (48 women and 36 men; mean age 37.4 ± 8.1 years) who completed the study were reported in a previous article [22]. No significant differences in baseline characteristics were observed between the treatment groups (Table 1, Table 2 and Table 3). 

### 3.2. Diet 

Adherence to diet was good (S = 122 ± 32, E = 114 ± 24, SE = 104 ± 34, C = 97 ± 17%; NS). All groups significantly reduced their energy intake compared to baseline (*p* < 0.01), with no significant differences between groups (Table 1). According to their energy profiles, all subjects approached the recommendations of the dietary intervention, increasing significantly the percentage of energy provided by carbohydrate (*p* < 0.05) and decreasing significantly the percentage of energy provided by fat (*p* < 0.05). Cholesterol intake decreased significantly by the end of the intervention period in the C group (*p* < 0.05). At baseline, all subjects had a diet “needing improvement”, however at the end of the intervention all groups improved their HEI index (*p* < 0.05), approaching “good”.

### 3.3. Physical Activity

The training programs adherence was adequate (S = 92.3 ± 4.0, E = 91.2 ± 4.9, SE = 92.5 ± 4.3%; NS). Time spent sitting per week decreased significantly in all groups after intervention with no significant differences between groups (Table 2). In addition, all groups increased their total physical activity, except C group (724 ± 1979 MET-min/week). After the intervention, all groups increased the percentage of “high physical activity”, more so in the S, E, and SE groups. All groups had a significant increase in VO2peak after intervention with no difference between them (Table 2).

### 3.4. Weight and Body Composition

After 22 weeks, a significant reduction in BMI, WC, and TFM was detected in all groups (*p* < 0.001), with no significant differences between them (Table 3). However, a post-hoc correction revealed that the change in TFM and TLM was greater in the SE group compared with the C group (*p* < 0.05). Body weight changes in the SE group showed a trend towards significance with respect to the S group (*p* = 0.059). 

The datasets used and/or analysed during the current study are available from the corresponding author upon reasonable request.

## 4. Discussion

The major finding of this work is that the SE exercise protocol while following a balanced, individualized, hypocaloric diet had a greater effect on the reduction of total fat mass than other exercise protocols. The subject’s behaviour also changed. All groups showed a dietary quality improvement moving from “needing improvement” at baseline to almost “good” at the end of the intervention. In fact, all intervention groups showed an increase in carbohydrate intake to the detriment of lipid intake, actions associated with weight loss [12,30,31].

Earlier reports have suggested that combined training can lead to greater reductions in body fat and metabolic improvements than other types of exercise, although this depends largely on the way exercises are combined [32,33]. Other studies have concluded that dietary restriction combined with endurance training is effective in achieving weight loss and reducing fat mass in obese subjects [17,18,34]. Few studies, however, have compared the effects of equal amounts of aerobic and resistance exercise (intensity of and volume of the training) on body mass and fat mass in overweight people. The present data show that combining aerobic and resistance exercises (SE) along with a balanced, individualized hypocaloric diet is more effective in promoting fat loss than following a hypocaloric diet and physical activity recommendations or any other type of exercise. This may be explained in that, while the intensity and volume of exercise performed by each group was designed to be equal, the actual energy expenditure of each group may have differed. In this regard, previous work has shown that a combined exercise protocol increases energy expenditure with less perceived exertion [35]. This might help subjects adhere to the SE exercise program, an added advantage when trying to reduce body fat [36]. In a previous PRONAF study of obese subjects following the same protocols as used in the present work, no significant differences were seen between groups in terms of changes in anthropometric and body composition variables [13]. In this regard, it may be that SE overweight subjects of the present study, which are closer to their ideal weight than the above obese subjects, found their exercise routines less demanding and therefore adhered better to them. 

The changes recorded in TFM ranged from 3.94% (group C) to 6.74% (group SE) (Table 3) and were greater than the ~2% reported by Skrypnik et al. [37], and even the ~5% reported in the above study on obese subjects [13]. Other authors also refer to greater changes in TFM in overweight than in obese subjects [38,39,40], probably due to the greater fitness of the former. Previous studies report that intensity can play an important role in long term weight loss programs, as well as weight bearing training, to prevent the plateau effect that usually occurs [12].

In recent years, interest has grown in the influence of lean mass (LM) on weight loss [17,41]. The maintenance of LM appears crucial in slowing future weight regain since it helps conserve the resting energy expenditure achieved with weight loss. It is well known that exercise has an important role in maintaining LM [17,42], although the effect is greater on the water content of the muscle than on the actual structural proteins [43]. In this regard, the increase in the percentage of “high physical activity” subjects (Table 2) may have been key in the maintenance of mean TLM seen in the SE group (Table 3). 

A trial limitation is that we did not analyse the effects of an individualised, balanced, hypercaloric diet alone from the effects of diet plus exercise. However, the international recommendations suggest exercise and diet as the ideal treatment to control excess body weight.

## 5. Conclusions

In conclusion, when keeping to an individualised, balanced, hypercaloric diet, some types of exercise may have a greater effect on weight loss and the reduction of body fat. The present results suggest that SE exercise in combination with a balanced, individualized hypocaloric diet may be the most recommendable protocol for the management of weight loss and fat mass in overweight subjects. 

## Figures and Tables

**Table 1:** Dietetic variables at baseline and at the end of intervention (Mean ± SD). N = 84, 48 women/36 men.

Group/Characteristics	S (*n* = 19)	E (*n* = 25)	SE (*n* = 22)	C (*n* = 18)	ANOVA
Energy Intake (kcal/d)					
baseline	2062 ± 477	2050 ± 432	2196 ± 410	2345 ± 545	NS
final	1500 ± 226 **	1572 ± 343 ***	1848 ± 420 ***	1763 ± 236 **	
change	−562 ± 516	−478 ± 314	−348 ± 478	−582 ± 512	NS
Energy profile					
Proteins (%EI)					
baseline	17.23 ± 2.53	17.01 ± 2.85	16.13 ± 2.52	20.63 ± 11.05	NS
final	18.82 ± 2.1 *	19.12 ± 3.17 *	18.56 ± 2.32 **	20.08 ± 2.44	
change	1.59 ± 2.57	2.12 ± 3.12	2.43 ± 3.23	−0.55 ± 10.03	NS
Carbohydrates (%EI)					
baseline	39.79 ± 6.81	36.76 ± 4.51	39.87 ± 5.78	34 ± 8.01	NS
final	44.44 ± 6.76 *	45.7 ± 8.02 ***	43.21 ± 4.84 *	41.91 ± 4.54 *	
change	4.66 ± 8.76	8.94 ± 8.34	3.35 ± 5.78	7.91 ± 8.81	NS
Lipids (%EI)					
baseline	38.2 ± 6.27	42.52 ± 4.64	39.93 ± 6.58	41.76 ± 7.48	NS
final	33.3 ± 6.31 *	30.88 ± 8.46 ***	35.06 ± 3.75 **	34.56 ± 4.79 *	
change	−4.9 ± 8.72	−11.64 ± 9.77	−4.87 ± 5.74	−7.2 ± 8.11	0.056
Alcohol (%EI)					
baseline	2.16 ± 3.22	1.35 ± 2.19	1.83 ± 3.0	1.45 ± 2.5	NS
final	0.55 ± 1.0 *	0.61 ± 2.3	0.84 ± 1.38	1.2 ± 2.2	
change	−1.61 ± 2.85	−0.73 ± 1.61	−1.0 ± 3.29	−0.25 ± 5.40	NS
Fibre (g/d)					
baseline	17.59 ± 7.47	17.26 ± 5.5	20.01 ± 4.42	20.61 ± 4.22	NS
final	17.75 ± 4.47	21.01 ± 5.58*	20.74 ± 6.73	19.55 ± 3.75	
change	0.16 ± 6.8	3.74 ± 7.05	0.73 ± 6.82	−1.06 ± 5.88	NS
Cholesterol (mg/d)					
baseline	308.0 ± 96	282.73 ± 103.01	322.59 ± 102.51	384.82 ± 121.31	NS
final	250.67 ± 120.5	231.37 ± 89.06	274.29 ± 99.85	259.36 ± 92.47 *	
change	−57.33 ± 153.7	−51.36 ± 126.03	−48.29 ± 156.55	−125.4 ± 153.2	NS
HEI					
baseline	59.8 ± 15.9	54.4 ± 9.5	56.07 ± 12.92	54.5 ± 10.1	NS
final	71.74 ± 7.18 *	81.4 ± 4.88 *	75.30 ± 4.22 *	72.8 ± 8.18 *	
change	11.94 ± 22.53	27.0 ± 12.95	19.23 ± 18.08	18.3 ± 7.8	NS

S = Strength group; E = Endurance group; SE = Combined strength and endurance group; C = Control group; EI = Energy Intake; HEI = Healthy Eating Index; Significantly different compared to baseline (* *p* < 0.05; ** *p* < 0.01; *** *p* < 0.001). NS = Not significantly different.

**Table 2:** Physical activity and training variables at baseline and at the end of intervention (Mean ± SD). N = 84, 48 women/36 men.

Group /Characteristics	S (*n* = 19)			E (*n* = 25)			SE (*n* = 22)			C (*n* = 18)			ANOVA
Sitting time (h/week)													
baseline	7.33 ± 4.85			6.98 ± 3.07			7.94 ± 3.59			8.07 ± 4.08			NS
final	5.78 ± 3.55 *			5.52 ± 2.40 *			6.21 ± 3.25 *			6.67 ± 3.27 *			
change	−1.55 ± 3.04			−1.46 ± 2.92			−1.73 ± 2.36			−1.40 ± 3.22			NS
Total PhA (MET-min/week)													
baseline	1331 ± 945			1022 ± 767			1609 ± 2016			1747 ± 1789			NS
final	2481 ± 1474 **			2647 ± 2070 ***			3308 ± 1816 **			2471 ± 1700			
change	1159 ± 1740			1625 ± 1790			1699 ± 2516			724 ± 1979			NS
Physical activity categories (% subjects)	Low	Medium	High	Low	Medium	High	Low	Medium	High	Low	Medium	High	
baseline	14.3	66.7	19	40	56	4	43.5	43.5	13	31.6	42.1	26.3	
final	23.8	9.5	66.7	4	24	72	13	13	73.9	42.1	26.3	31.6	
change	9.5	−57.2	47.7#	−36	−32	68#	−30.5	−27.5	60.9#	10.4	−15.8	5.3#	<0.05
VO2peak (mL/min)													
baseline	2428 ± 728			2420 ± 635			2725 ± 802			2468 ± 720			NS
final	2707 ± 932 **			2745 ± 655***			3134 ± 888 ***			2758 ± 770 **			
change	279 ± 399			321 ± 243			409 ± 421			289 ± 400			NS

S = Strength group; E = Endurance group; SE = Combined strength and endurance group; C = Control group; PhA = Physical Activity. Significant differences compared with the baseline (* *p* < 0.05; ** *p* < 0.01; *** *p* < 0.001). NS = Not significantly different. Significant differences between the changes obtained in each group (Bonferroni post hoc correction: # *p* < 0.05).

**Table 3:** Body composition variables at baseline and at the end of intervention (Mean ± SD). N = 84, 48 women/36 men.

Group/Characteristics	S (*n* = 19)	E (*n* = 25)	SE (*n* = 22)	C (*n* = 18)	ANOVA
Body weight (kg)					
Baseline	79.4 ± 10.3	79.5 ± 10.6	80.2 ± 11.2	79.8 ± 10.2	NS
Final	74.8 ± 11.1 ***	72.9 ± 10.4 ***	71.7 ± 10.8 ***	74.4 ± 10.1 ***	
Change	−4.6 ± 4.5#	−6.6 ± 4.6	−8.5 ± 2.8#	−6.1 ± 5.6	0.059
BMI (kg/m^2^)					
Baseline	28.56 ± 1.1	28.64 ± 1.46	27.74 ± 1.12	28.24 ± 1.35	NS
Final	26.37 ± 1.13 ***	25.9 ± 1.56 ***	25.12 ± 1.69 ***	26.11 ± 1.93 ***	
Change	−2.19 ± 1.04	−2.74 ± 1.55	−2.62 ± 1.26	−2.13 ± 1.6	NS
WC (cm)					
Baseline	95.54 ± 5.1	95.84 ± 8.34	94.34 ± 6.61	93.20 ± 5.56	NS
Final	87.14 ± 5.15 ***	87.11 ± 7.99 ***	84.73 ± 6.41 ***	86.91 ± 6.52 ***	
change	−8.39 ± 3.98	−8.73 ± 3.55	−9.61 ± 3.64	−6.29 ± 5.36	NS
TFM (%)					
Baseline	40.56 ± 7.48	39.33 ± 5.47	36.36 ± 6.31	40.34 ± 5.18	NS
Final	36.32 ± 7.77 ***	34.59 ± 6.36 ***	29.62 ± 8.11 ***	36.39 ± 6.31 **	
Change	−4.24 ± 2.02	−4.74 ± 2.96	−6.74 ± 3.27#	−3.94 ± 4.18#	<0.05
AF (%)					
Baseline	46.77 ± 7.50	44.06 ± 7.36	42.35 ± 5.56	46.02 ± 6.82	NS
Final	41.24 ± 8.47 ***	36.93 ± 8.82 ***	32.05 ± 9.26 ***	39.96 ± 8.05 **	
Change	−5.53 ± 3.83	−7.13 ± 5.79	−10.3 ± 6.26	−6.06 ± 7.05	NS
AF/GF					
Baseline	1.11 ± 0.18	1.07 ± 0.17	1.19 ± 0.28	1.088 ± 0.21	NS
Final	1.09 ± 0.17	1.01 ± 0.2 **	1.08 ± 0.26 ***	1.02 ± 0.18 *	
Change	−0.03 ± 0.09	−0.06 ± 0.09	−0.11 ± 0.11	−0.06 ± 0.12	NS
TLM (%)					
Baseline	43.41 ± 9.24	45.75 ± 8.4	47.68 ± 8.83	46.62 ± 7.98	NS
Final	43.45 ± 9.75	45.24 ± 8.33	47.98 ± 9.22	45.68 ± 7.61 *	
Change	0.03 ± 0.9	−0.5 ± 1.24	0.3 ± 1.52#	−0.94 ± 1.59#	<0.05
DMO (T-Score)					
Baseline	0.71 ± 0.8	0.46 ± 0.85	0.34 ± 0.91	0.08 ± 0.9	NS
Final	0.87 ± 0.8 ***	0.64 ± 0.86 **	0.58 ± 0.79 ***	0.25 ± 0.92 ***	
Change	0.17 ± 0.17	0.18 ± 0.2	0.24 ± 0.18	0.17 ± 0.11	NS

S = Strength group; E = Endurance group; SE = Combined strength and endurance group; C = Control group. BMI = Body Mass Index; WC = Waist Circumference; TFM = Total Fat Mass; AF = Android Fat; AF/GF = Android/Gynoid Ratio; TLM = Total Lean Mass. DMO = Densitometry. Significant differences intragroup compared with the baseline (* *p* < 0.05; ** *p* < 0.01; *** *p* < 0.001). NS = Not significantly different. Significant differences between the changes obtained in each group (Bonferroni post hoc correction: # *p* < 0.05).
